# Stronger Quantum Speed Limit for Mixed Quantum States

**DOI:** 10.3390/e25071046

**Published:** 2023-07-12

**Authors:** Shrobona Bagchi, Dimpi Thakuria, Arun Kumar Pati

**Affiliations:** 1 Center for Quantum Information, Korea Institute of Science and Technology, Seoul 02792, Republic of Korea; 2 Quantum Information and Computation Group, Harish-Chandra Research Institute, Chhatnag Road, Jhunsi, Allahabad 211019, India; 3 Homi Bhabha National Institute, Anushaktinagar, Training School Complex, Mumbai 400085, India

**Keywords:** quantum speed limit, mixed quantum states, time-energy uncertainty relation

## Abstract

In this paper, we derive a quantum speed limit for unitary evolution for the case of mixed quantum states using the stronger uncertainty relation for mixed quantum states. This bound can be optimized over different choices of Hermitian operators for a better bound. We illustrate this with some examples and show its better performance with respect to three existing bounds for mixed quantum states.

## 1. Introduction

Uncertainty relations have been of fundamental importance in quantum mechanics since the birth of quantum mechanics in the early nineties. The uncertainty principle was first proposed by Werner Heisenberg heuristically [1]. He provided a lower bound to the product of standard deviations of the position and the momentum [1] of a quantum particle. Not only this, the uncertainty relations are also capable of capturing the intrinsic restrictions in the preparation of quantum systems, which are termed as the preparation uncertainty relations [2]. In this direction, Robertson formulated the so-called preparation uncertainty relation for two arbitrary quantum-mechanical observables, which are generally non-commuting [2]. However, the Robertson uncertainty relation does not completely express the incompatible nature of two non-commuting observables in terms of uncertainty quantification and is not the most optimal nor the most tight one. It also suffers from the triviality problem of uncertainty relations. To improve on these deficiencies, the stronger variations of the uncertainty relations have been proven which capture the notion of incompatibility more efficiently and also provide an improved lower bound on the sum and product of variances of the generally incompatible observables [3,4]. On another note, and along the same lines of the formulation of uncertainty relations, the energy–time uncertainty relation [5,6] proved to be quite different from the preparation uncertainty relations of other observables, such as the position and momentum or that of the angular momentum because time is not treated as an operator in quantum mechanics [7]. Thus, time not being a quantum observable, the time–energy uncertainty relation lacked a good interpretation such as for those of the other quantum mechanical observables such as position and momentum. Mandelstam and Tamm derived an uncertainty relation [8] which is now called an energy–time uncertainty relation. It follows from the Robertson uncertainty relation when we consider the initial quantum state and the Hamiltonian as the corresponding quantum mechanical operators [8] and Δt as the time interval between the initial and final state after the evolution. An interpretation of this time energy uncertainty relation was given in terms of the so-called quantum speed limit [5,6]. In the current literature, there are several other approaches to obtain quantum speed limits for closed quantum system dynamics [9,10,11,12,13,14,15,16,17,18,19,20,21,22,23,24,25,26,27,28,29,30,31,32,33,34,35,36,37,38,39,40,41,42,43,44,45,46,47,48] as well as for open quantum system dynamics [49,50,51,52,53,54,55,56,57,58,59]. Quantum speed limits have also been generalised to the cases of the arbitrary evolution of quantum systems [60], unitary operator flows [61], change of bases [62], and for the cases of arbitrary phase spaces [63]. Most recently, in another direction, exact quantum speed limits have also been proposed [64].

The notion of a quantum speed limit is not only of fundamental importance, but also has many practical applications in quantum information, computation, and communication technology. Quantum speed limit bounds have proven to be very useful in quantifying the maximal rate of quantum entropy production [65,66], the maximal rate of quantum information processing [57,67], quantum computation [68,69,70] in optimal control theory [71,72], quantum thermometry [73], and quantum thermodynamics [74]. These explorations motivate us to find better quantum speed limit bounds that can go beyond the existing bounds in the literature. In this paper, we use the stronger uncertainty relation developed in [3], then generalise to the case of mixed quantum states to derive a stronger form of quantum speed limit for mixed quantum states undergoing unitary evolution. We show that the new bound provides a stronger expression of quantum speed limit compared to the MT-like bound for mixed quantum states. This bound can also be optimized over many operators. We then find various examples for mixed states and some example Hamiltonians that shows the better performance of our bound over the MT-like bound for mixed quantum states and the bounds for mixed states in Ref. [41].

The present article is organised as follows. In Section 2.1 and Section 2.2, we give a background that includes the various forms of quantum speed limit for mixed quantum states (Section 2.1), followed by the stronger uncertainty relations for mixed quantum states in Section 2.2. In Section 3, we derive the stronger quantum speed limit for mixed quantum states, respectively, and show methods to calculate the set of operators obeying a necessary condition for the bound to hold true. In Section 4.1, we show its better performance with examples of random Hamiltonians, specific examples of Hamiltonians that are useful in quantum computation, and random quantum states, respectively, over three different previous bounds of quantum speed limit for mixed quantum states. Finally, in Section 5 we conclude and point to future directions.

## 2. Background

### 2.1. Quantum Speed Limits

Quantum speed limit is one of the interpretations of the time–energy uncertainty relation in quantum mechanics. In particular, Mandelstam and Tamm derived the first expression of the quantum speed limit time as $\tau_{QSL}=\frac{\pi }{2\Delta H}$, where ΔH is the variance of the Hamiltonian driving the quantum system *H* [8]. As an interpretation of their bound, they also argued that $\tau_{QSL}$ quantifies the life-time of quantum states. Their interpretation was further solidified by Margolus and Levitin [75], who derived an alternative expression for $\tau_{QSL}$ in terms of the expectation value of the Hamiltonian as $\tau_{QSL}=\frac{\pi }{2\langle H\rangle }$. Eventually, it was also shown that the combined bound,

(1) $$\begin{array}{c} \tau_{QSL}=\max\left\{\frac{\pi \hbar }{2\Delta H},\frac{\pi \hbar }{2\langle H\rangle }\right\} \end{array}$$

is tight. Many more versions of quantum speed limits have been proposed since then, with an intent to improve the previous bounds in terms of tightness and performance. In this direction, recently a stronger quantum speed limit for the pure quantum states has been proposed as follows.

(2) $$\begin{array}{c} \tau \geq \frac{\hbar s_{0}}{2\Delta H}+\int_{0}^{\tau }R\left(t\right)dt, \end{array}$$

where we have

(3) $$\begin{array}{c} R\left(t\right)=\frac{1}{2}|\langle \Psi^{⊥}\left(t\right)|\frac{A}{\Delta A}\pm i\frac{H}{\Delta H}{\left|\Psi \left(t\right)\rangle \right|}^{2}. \end{array}$$

The stronger quantum speed limit bound generally performs better than the MT bound for pure quantum states since it can be shown that for pure quantum states R(t)≥0 in general. On the other hand, quantum speed limits for the mixed quantum states have also been proposed in various forms [41]. Quantum speed limit can be extended to the case of mixed quantum states by defining the distance between the initial state $\rho_{0}$ and the final state $\rho_{t}$ as their Bures angle $L\left(\rho_{0},\rho_{t}\right)=\arccos\left(F\left(\rho_{0},\rho_{t}\right)\right)$, with $F\left(\rho_{0},\rho_{t}\right)=\mathrm{tr}\left[\sqrt{\sqrt{\rho_{0}}\rho_{t}\sqrt{\rho_{0}}}\right]$ being the Uhlmann root fidelity,

(4) $$\begin{array}{c} \tau_{L}=\frac{L(\rho_{0},\rho_{t})}{min\{H,\Delta H\}}, \end{array}$$

where ℏ=1 has been set for convenience. It bounds the evolution time required to evolve the mixed state $\rho_{0}$ to the final state $\rho_{t}$ by means of a unitary operator $U_{t}$, i.e., $\rho_{t}=U_{t}\rho_{0}U_{t}^{†}$, where the quantum system is governed by a time-dependent Hamiltonian $H_{t}$. There are many other forms of speed limits for mixed quantum states, which we leave for later investigation in future research. In [41] another bound tighter than the MT bound was derived for the speed of unitary evolution. According to this bound, the minimum time required to evolve from state ρ to state σ by means of a unitary operation generated by the Hamiltonian $H_{t}$ is bounded from below by

(5) $$\begin{array}{c} T_{\Theta }\left(\rho ,\sigma \right)=\tau_{2}=\frac{\Theta (\rho ,\sigma )}{Q_{\Theta }}\;\;\;\mathrm{where} \end{array}$$

(6) $$\begin{array}{c} Q_{\Theta }=\frac{1}{T}\int_{0}^{T}dt\sqrt{\frac{2\mathrm{Tr}(\rho_{t}^{2}H_{t}^{2}-{\left(\rho_{t}H_{t}\right)}^{2})}{\mathrm{Tr}(\rho_{t}^{2}-\frac{1}{N^{2}})}}\;\;\;\mathrm{and} \end{array}$$

(7) $$\begin{array}{c} \Theta \left(\rho ,\sigma \right)=\arccos\sqrt{\frac{\left(\mathrm{Tr}\left(\rho \sigma \right)-\frac{1}{N}\right)}{\left(\mathrm{Tr}\left(\rho^{2}\right)-\frac{1}{N}\right)}} \end{array}$$

where *N* is the dimension of the quantum system undergoing unitary evolution due to the time-independent Hamiltonian *H*. We mention this bound since this bound does not reduce to the MT bound in general. However, there is another bound proposed in the same paper that reduces to the MT bound for the case of pure states. It is given as follows:

(8) $$\begin{array}{c} T_{\Phi }\left(\rho ,\sigma \right)=\tau_{2}=\frac{\Phi (\rho ,\sigma )}{Q_{\Phi }}\;\;\;\mathrm{where} \end{array}$$

(9) $$\begin{array}{c} Q_{\Phi }=\frac{1}{T}\int_{0}^{T}dt\sqrt{\frac{\mathrm{Tr}(\rho_{t}^{2}H_{t}^{2}-{\left(\rho_{t}H_{t}\right)}^{2})}{\mathrm{Tr}(\rho_{t}^{2})}}\;\;\;\mathrm{and} \end{array}$$

(10) $$\begin{array}{c} \Phi \left(\rho ,\sigma \right)=\arccos\sqrt{\frac{\mathrm{Tr}(\rho \sigma )}{\mathrm{Tr}(\rho^{2})}} \end{array}$$

We work with these different quantum speed limits for mixed quantum states and point out some examples where the newly derived quantum speed limit bound for mixed quantum states here performs better than the above bounds.

### 2.2. Stronger Uncertainty Relations for General Mixed Quantum States

Robertson gave rigorous and quantitative formulations of the heuristic Heisenberg’s uncertainty principle, which are called the preparation uncertainty relations [2]. This is stated as the following. For any two non-commuting operators A and B, the Robertson–Schroedinger uncertainty relation for the state of the system |Ψ〉 is given by the following inequality:

(11) $$\begin{array}{c} \Delta A^{2}\Delta B^{2}\geq {\left|\frac{1}{2}\langle [A,B]\rangle \right|}^{2}+{\left|\frac{1}{2}\langle \left\{A,B\right\}\rangle -\langle A\rangle \langle B\rangle \right|}^{2}, \end{array}$$

where the averages and the variances are defined over the state of the quantum system ρ. However, this uncertainty bound is not optimal. There have been several attempts to improve the bound. Here, we state a stronger bound obtained from an alternative uncertainty relation also called the Maccone–Pati uncertainty relation [3] and it is also state dependent.

(12) $$\begin{array}{c} \Delta A\Delta B\geq \frac{i}{2}\frac{\mathrm{Tr}(\rho [A,B])}{\left(1-\frac{1}{2}|\mathrm{Tr}\left(\rho^{\frac{1}{2}}\left(\frac{A}{\Delta A}\pm i\frac{B}{\Delta B}\right)\sigma \right){|}^{2}\right)}, \end{array}$$

where $\mathrm{Tr}(\rho^{\frac{1}{2}}\sigma )=0$ and ${\left||\sigma |\right|}_{2}=1$. This uncertainty relation has been proved to be stronger than the Robertson–Schrodinger uncertainty relation. It is optimized to an equality when maximized over all possible σ, such that we have the optimized bound as

(13) $$\begin{array}{c} \Delta A\Delta B\geq \max_{\sigma }\frac{i}{2}\frac{\mathrm{Tr}(\rho [A,B])}{\left(1-\frac{1}{2}|\mathrm{Tr}\left(\rho^{\frac{1}{2}}\left(\frac{A}{\Delta A}\pm i\frac{B}{\Delta B}\right)\sigma \right){|}^{2}\right)}. \end{array}$$

We can take the absolute values on both sides and then perform optimization, so that we get the following uncertainty relation:

(14) $$\begin{array}{c} \Delta A\Delta B\geq \max_{\sigma }\frac{1}{2}\frac{\left|\mathrm{Tr}(\rho [A,B])\right|}{\left|(1-\frac{1}{2}|\mathrm{Tr}\left(\rho^{\frac{1}{2}}\left(\frac{A}{\Delta A}\pm i\frac{B}{\Delta B}\right)\sigma \right){|}^{2})\right|}. \end{array}$$

We will use the above stronger uncertainty relations for mixed quantum states to derive a stronger version of quantum speed limits for mixed quantum states. See [76] for the proof of the stronger uncertainty relations for mixed quantum states.

## 3. Result: Stronger Quantum Speed Limit for Unitary Evolution

In this section, we prove a stronger version of quantum speed limits for mixed quantum states using the stronger uncertainty relations for mixed quantum states. The theorem on stronger quantum speed limit for mixed quantum states is stated in the following paragraph.

**Theorem** **1.** *The time evolution of a general mixed quantum state governed by a unitary operation generated by a Hamiltonian is given by the following equation:*

(15) $$\begin{array}{c} \tau \geq \tau_{SQSLM}=\frac{\sqrt{\mathrm{Tr}(\rho_{0}^{2})}}{2\Delta H}\times \\ \int_{s_{0}\left(0\right)}^{s_{0}\left(\tau \right)}\frac{\sin s_{0}\left(t\right)}{\left(1-R\left(t\right)\right)\cos\frac{s_{0}\left(t\right)}{2}\sqrt{\left(1-\mathrm{Tr}\left(\rho_{0}^{2}\right)\cos^{2}\frac{s_{0}\left(t\right)}{2}\right)}}ds_{0}, \end{array}$$

*where $\tau_{SQSLM}$ stands as a short form for the stronger quantum speed limit for mixed quantum states and we have the following definitions of the quantities expressed in the above equation:*

$$\begin{array}{c} s_{0}\left(t\right)=2\cos^{-1}\left|\sqrt{\frac{\mathrm{Tr}(\rho (0)\rho (t))}{\mathrm{Tr}(\rho_{0}^{2})}}\right|,\\ \Delta H=\mathrm{Tr}\left(H^{2}\rho \right)-{\left(\mathrm{Tr}\left(H\rho \right)\right)}^{2}\\ R\left(t\right)=\frac{1}{2}{\left|\mathrm{Tr}\left(\rho^{\frac{1}{2}}\left(\frac{A}{\Delta A}\pm i\frac{B}{\Delta B}\right)\sigma \right)\right|}^{2},\\ \mathrm{where}\;\mathrm{Tr}\left(\rho^{\frac{1}{2}}\sigma \right)={0\;\mathrm{and}\;||\sigma ||}_{2}=1, \end{array}$$

*where we have denoted $\rho_{0}=\rho \left(0\right)$, ρ=ρ(t) and used this interchangeably everywhere, ${\left||\sigma |\right|}_{2}={\left(\sum_{n\in I}\langle e_{n}|\sigma \sigma^{†}|e_{n}\rangle \right)}^{\frac{1}{2}}$, $\left\{|e_{n}\rangle \right\}$ forming a complete orthonormal basis in Hilbert space H, $\sigma \in L^{2}\left(H\right)$, i.e., σ belongs to the set of all Hilbert Schmidt linear operators.*

**Proof of Theorem 1.**  The proof of the above theorem goes as follows. We start by writing out the stronger uncertainty relation for mixed quantum states as is given by the following:

(16) $$\begin{array}{c} \Delta A\Delta B\geq \frac{1}{2}\frac{\left|\mathrm{Tr}(\rho [A,B])\right|}{\left|(1-\frac{1}{2}|\mathrm{Tr}\left(\rho^{\frac{1}{2}}\left(\frac{A}{\Delta A}\pm i\frac{B}{\Delta B}\right)\sigma \right){|}^{2})\right|}, \end{array}$$

See [76] for the derivation of the above inequality. From the stronger uncertainty relation for mixed quantum states, we get the following:

(17) $$\begin{array}{c} \Delta A\Delta H\left(1-R\left(t\right)\right)\geq \frac{1}{2}\left|\mathrm{Tr}\left(\rho \left[A,H\right]\right)\right|, \end{array}$$

where we have defined R(t) as the following:

(18) $$\begin{array}{c} R\left(t\right)=\frac{1}{2}{\left|\mathrm{Tr}\left(\rho^{\frac{1}{2}}\left(\frac{A}{\Delta A}\pm i\frac{B}{\Delta B}\right)\sigma \right)\right|}^{2} \end{array}$$

and have taken $A=\rho_{0}$ and B=H for our purpose of deriving the stronger quantum speed limit for mixed quantum states. This particular choice of these operators helps us to formulate our inequality for the quantum speed limit for mixed quantum states. Additionally, for mixed quantum states, from Eahrenfest’s theorem we get the following:

(19) $$\begin{array}{c} i\hbar \frac{d\mathrm{Tr}(\rho A)}{dt}=\mathrm{Tr}\left(\rho \left[A,H\right]\right) \end{array}$$

Therefore, from the above equations, we get the following:

(20) $$\begin{array}{c} \Delta A\Delta H\left(1-R\left(t\right)\right)\geq \frac{\hbar }{2}\left|\frac{d\langle A\rangle }{dt}\right| \end{array}$$

The variance of the operator *A* is then given by

(21) $$\begin{array}{cc} \Delta A^{2} & =\mathrm{Tr}\left(\rho {\left(0\right)}^{2}\rho \left(t\right)\right)-{\left(\mathrm{Tr}\left(\rho \left(0\right)\rho \left(t\right)\right)\right)}^{2}\\ & =\mathrm{Tr}\left(\rho_{0}^{2}\rho_{t}\right)-{\left(\mathrm{Tr}\left(\rho_{0}\rho_{t}\right)\right)}^{2}, \end{array}$$

where we have used the notation $\rho \left(0\right)=\rho_{0}$ and $\rho \left(t\right)=\rho_{t}$. We can now take the following parametrization:

(22) $$\begin{array}{c} \langle A\rangle =\mathrm{Tr}\left(\rho \left(0\right)\rho \left(t\right)\right)=\mathrm{Tr}\left(\rho_{0}^{2}\right)\cos^{2}\frac{s_{0}\left(t\right)}{2}. \end{array}$$

Now, using the equation of motion for the average of *A*

$$\begin{array}{c} |\hbar \frac{d}{dt}\langle A\rangle |=|\langle \left[A,H\right]\rangle |, \end{array}$$

where the averages are all with respect to the mixed quantum state ρ and the quantum mechanical hermitian operator *A* has no explicit time dependence. Thus, using Equation (22), we get

(23) $$\begin{array}{c} |\frac{d\langle A\rangle }{dt}|=\mathrm{Tr}\left(\rho_{0}^{2}\right)\frac{\sin s_{0}\left(t\right)}{2}\frac{ds_{0}}{dt} \end{array}$$

Now, let us analyze the structure of $\Delta A^{2}$ as follows:

(24) $$\begin{array}{c} \Delta A^{2}=\mathrm{Tr}\left(\rho_{0}^{2}\rho_{t}\right)-{\left(\mathrm{Tr}\left(\rho_{0}\rho_{t}\right)\right)}^{2}. \end{array}$$

Let {|k〉} be the eigenbasis from the singular value decomposition of the density matrix $\rho_{0}$. Then, we have the following expression:

(25) $$\begin{array}{c} \rho_{0}=\sum_{k}\lambda_{k}|k\rangle \langle k|\;\mathrm{and}\;\rho_{0}^{2}=\sum_{k}\lambda_{k}^{2}|k\rangle \langle k|. \end{array}$$

Using the above equation we obtain the following quantities:

(26) $$\begin{array}{c} \mathrm{Tr}\left(\rho_{0}\rho_{t}\right)=\sum_{k}\lambda_{k}\langle k|\rho_{t}|k\rangle \;\mathrm{and}\;\mathrm{Tr}\left(\rho_{0}^{2}\rho_{t}\right)=\sum_{k}\lambda_{k}^{2}\langle k|\rho_{t}|k\rangle . \end{array}$$

Since we know that $0\leq \lambda_{k}^{2}\leq \lambda_{k}\leq 1\;\forall \;k$ and also $\langle k|\rho_{t}|k\rangle \geq 0\;\forall \;k$ because $\rho_{t}$ is a positive operator. Therefore, we get the following inequality:

(27) $$\begin{array}{c} \mathrm{Tr}\left(\rho_{0}\rho_{t}\right)\geq \mathrm{Tr}\left(\rho_{0}^{2}\rho_{t}\right). \end{array}$$

Adding $-{\left(\mathrm{Tr}\left(\rho_{0}\rho_{t}\right)\right)}^{2}$ on both side of the above equation we get

(28) $$\begin{array}{cc} \mathrm{Tr}\left(\rho_{0}\rho_{t}\right)-{\left(\mathrm{Tr}\left(\rho_{0}\rho_{t}\right)\right)}^{2} & \geq \mathrm{Tr}\left(\rho_{0}^{2}\rho_{t}\right)-{\left(\mathrm{Tr}\left(\rho_{0}\rho_{t}\right)\right)}^{2}\\ \; & =\Delta A^{2}. \end{array}$$

Now, using Equation (22) we get

(29) $$\begin{array}{c} \mathrm{Tr}\left(\rho_{0}^{2}\right)\cos^{2}\frac{s_{0}\left(t\right)}{2}\left(1-\mathrm{Tr}\left(\rho_{0}^{2}\right)\cos^{2}\frac{s_{0}\left(t\right)}{2}\right)\geq \Delta A^{2} \end{array}$$

Taking the square root on both sides and multiplying by ΔH we get

(30) $$\begin{array}{c} \sqrt{\mathrm{Tr}(\rho_{0}^{2})}\cos\frac{s_{0}\left(t\right)}{2}\sqrt{\left(1-\mathrm{Tr}\left(\rho_{0}^{2}\right)\cos^{2}\frac{s_{0}\left(t\right)}{2}\right)}\Delta H\geq \Delta A\Delta H. \end{array}$$

From here, we get the following:

$$\begin{array}{c} \sqrt{\mathrm{Tr}(\rho_{0}^{2})}\cos\frac{s_{0}\left(t\right)}{2}\sqrt{\left(1-\mathrm{Tr}\left(\rho_{0}^{2}\right)\cos^{2}\frac{s_{0}\left(t\right)}{2}\right)}\Delta H\left(1-R\left(t\right)\right)\\ \geq \Delta A\Delta H(1-R(t)), \end{array}$$

since (1−R(t)) is a positive quantity here. From the previous equations we get the following:

$$\begin{array}{c} \sqrt{\mathrm{Tr}(\rho_{0}^{2})}\cos\frac{s_{0}\left(t\right)}{2}\sqrt{\left(1-\mathrm{Tr}\left(\rho_{0}^{2}\right)\cos^{2}\frac{s_{0}\left(t\right)}{2}\right)}\Delta H\left(1-R\left(t\right)\right)\\ \geq \Delta A\Delta H\left(1-R\left(t\right)\right)\geq \frac{\hbar }{2}\left|\frac{d\langle A\rangle }{dt}\right|=\mathrm{Tr}\left(\rho_{0}^{2}\right)\frac{\sin s_{0}\left(t\right)}{2}\frac{ds_{0}}{dt}, \end{array}$$

Therefore, from the above equations we get the following:

$$\begin{array}{c} \cos\frac{s_{0}\left(t\right)}{2}\sqrt{\left(1-\mathrm{Tr}\left(\rho_{0}^{2}\right)\cos^{2}\frac{s_{0}\left(t\right)}{2}\right)}\Delta H\geq \\ \frac{\sqrt{\mathrm{Tr}(\rho_{0}^{2})}}{\left(1-R(t)\right)}\frac{\sin s_{0}\left(t\right)}{2}\frac{ds_{0}}{dt}, \end{array}$$

Integrating the above equation with respect to *t* and *s* over their corresponding regions on both sides, we get for the case of time-independent Hamiltonian the following expression for quantum speed limit:

$$\begin{array}{c} \tau \geq \frac{\sqrt{\mathrm{Tr}(\rho_{0}^{2})}}{2\Delta H}\times \\ \int_{s_{0}\left(0\right)}^{s_{0}\left(\tau \right)}\frac{\sin s_{0}\left(t\right)}{\left(1-R\left(t\right)\right)\cos\frac{s_{0}\left(t\right)}{2}\sqrt{\left(1-\mathrm{Tr}\left(\rho_{0}^{2}\right)\cos^{2}\frac{s_{0}\left(t\right)}{2}\right)}}ds_{0}, \end{array}$$

where the definitions of the parametrizations have been stated in the statement of the theorem. One can also derive the quantum speed limit bound for mixed quantum states in a different way. Writing out the previous equations and rearranging terms on the right-hand side and the left-hand side in a different way, it can be shown that the quantum speed limit bound for the mixed quantum states can also be written following the procedure as stated below step-by-step. We start from the following inequality after rearranging the terms:

$$\begin{array}{c} \cos\frac{s_{0}\left(t\right)}{2}\sqrt{\left(1-\mathrm{Tr}\left(\rho_{0}^{2}\right)\cos^{2}\frac{s_{0}\left(t\right)}{2}\right)}\Delta H\left(1-R\left(t\right)\right)\geq \\ \frac{\sqrt{\mathrm{Tr}(\rho_{0}^{2})}\sin s_{0}\left(t\right)}{2}\frac{ds_{0}}{dt}, \end{array}$$

Integrating the above equation we get the following quantum speed limit bound for mixed quantum states:

$$\begin{array}{c} \tau \geq \\ \int_{s(0)}^{s(\tau )}\frac{\sqrt{\mathrm{Tr}(\rho_{0}^{2})}\sin s_{0}\left(t\right)}{2\Delta H\cos\frac{s_{0}\left(t\right)}{2}\sqrt{\left(1-\mathrm{Tr}\left(\rho_{0}^{2}\right)\cos^{2}\frac{s_{0}\left(t\right)}{2}\right)}}ds_{0}+\int_{0}^{\tau }R\left(t\right)dt, \end{array}$$

From the above equations, we get the following:

(31) $$\begin{array}{c} \tau \geq {\left[\frac{2\cos^{-1}\left(\sqrt{\mathrm{Tr}(\rho_{0}^{2})}\cos\frac{s_{0}}{2}\right)}{\Delta H}\right]}_{s(0)}^{s(\tau )}+\int_{0}^{\tau }R\left(t\right)dt \end{array}$$

Putting the values, we get the following equation for time-independent Hamiltonians:

(32) $$\begin{array}{c} \tau \geq \left[\frac{2(\cos^{-1}\left(\sqrt{\mathrm{Tr}(\rho_{0}\rho_{t})}\right)-\cos^{-1}\left(\sqrt{\mathrm{Tr}(\rho_{0}^{2})}\right))}{\Delta H}\right]\\ +\int_{0}^{\tau }R\left(t\right)dt \end{array}$$

It is easy to see that the above bound reduces to that of the stronger quantum speed limit bound for pure states when we take $\mathrm{Tr}(\rho_{0}^{2})=1$, which performs better than the MT bound for pure quantum states. □

### 3.1. Method to Find σ, Such That $\mathrm{Tr}(\rho^{\frac{1}{2}}\sigma )=$ 0

For the purpose of calculating our bound, we need to find ways to derive the structure of σ or identify the set of σ such that the condition $\mathrm{Tr}(\rho^{\frac{1}{2}}\sigma )=0$ is satisfied. In the preceding paragraphs, we found out two different ways to do so and will apply them to examples hereafter.

#### 3.1.1. Method I: ρ and σ ∈ Orthogonal Subspaces

In this section we derive a method that can be useful to find σ such that the condition $\mathrm{Tr}(\rho^{\frac{1}{2}}\sigma )=0$ holds. First, let us state the properties of σ that should be satisfied in that case. It should satisfy ${\left||\sigma |\right|}_{2}=1$, where ${\left||\sigma |\right|}_{2}={\left(\sum_{n\in I}\langle e_{n}|\sigma^{†}\sigma |e_{n}\rangle \right)}^{\frac{1}{2}}$ and $\sigma \in L^{2}\left(H\right)$. Let us take the following definitions:

(33) $$\begin{array}{c} \rho =\sum_{k}\lambda_{k}{\left|k\rangle \langle k|,\;\;\right|}^{\prime }\;\;\rho^{\frac{1}{2}}=\sum_{k}\lambda_{k}^{\frac{1}{2}}|k\rangle \langle k|, \end{array}$$

where we have $\sum_{k}\lambda_{k}=1$ fixed by the normalization constraint of ρ and we have taken the positive square root of $\lambda_{k}$. Note that we have written ρ in its eigenbasis and it can be reverted back to any other basis by unitary transformation and the same holds for $\rho^{\frac{1}{2}}$ in a corresponding way. In this way, $\rho^{\frac{1}{2}}$ is also a positive semidefinite Hermitian operator as ρ. Let us denote $\lambda_{k}^{\frac{1}{2}}=\eta_{k}$ for convenience. Therefore, following this notation, we have

(34) $$\begin{array}{c} \rho^{\frac{1}{2}}=\sum_{k}\eta_{k}|k\rangle \langle k|. \end{array}$$

Therefore from the condition $\mathrm{Tr}(\rho^{\frac{1}{2}}\sigma )=0$, we get

(35) $$\begin{array}{c} \mathrm{Tr}(\sum_{k}\eta_{k}|k\rangle \langle k|\sigma )=0. \end{array}$$

This translates to the following condition:

(36) $$\begin{array}{c} \sum_{k}\eta_{k}\langle k|\sigma |k\rangle =0. \end{array}$$

We know that $\eta_{k}\geq 0\;\forall \;k$ from our own constraint which we have specifically chosen that we only take the positive square root of $\lambda_{k}\;\forall \;k$ as $\eta_{k}$. Additionally, when we impose the condition that σ is also a positive operator, then we get the condition that 〈k|σ|k〉≥0∀k. One of the ways this condition can be obtained is that if ρ and σ are chosen from orthogonal subspaces. Let us note here that ρ is fixed here and we do not have a choice to fix ρ and we only have the freedom to choose any σ from the orthogonal subspace to that of ρ. As a result, we can optimize our bound for the stronger quantum speed limit over all possible choices of such σ chosen from the orthogonal subspaces to that of ρ. For mixed quantum states, this choice of σ becomes relevant only in higher dimensional Hilbert spaces than the qubit space.

#### 3.1.2. Method II: A Form of σ Written Directly in Terms of ρ and Hermitian Operators

There is another method that allows one to derive an operator that satisfies the condition $\mathrm{Tr}(\rho^{\frac{1}{2}}\sigma )=0$ in an easier way. This set of σ can be written down in the following form:

(37) $$\begin{array}{c} \sigma =\frac{O-\langle O\rangle }{\Delta O}\rho^{\frac{1}{2}}, \end{array}$$

where *O* is any Hermitian operator. This way, the conditions $\mathrm{Tr}(\rho^{\frac{1}{2}}\sigma )=0$ and $\mathrm{Tr}(\sigma \sigma^{†})=1$ are satisfied automatically. The proof of this claim in given in the following paragraph.

**Proof.**  The proof of the first condition $\mathrm{Tr}(\rho^{\frac{1}{2}}\sigma )=0$ goes as follows.

$$\begin{array}{c} \mathrm{Tr}\left(\rho^{\frac{1}{2}}\sigma \right)=\mathrm{Tr}\left(\rho^{\frac{1}{2}}\frac{O-\langle O\rangle }{\Delta O}\rho^{\frac{1}{2}}\right)=\frac{1}{\Delta O}\mathrm{Tr}\left(\rho \left(O-\langle O\rangle \right)\right)=0 \end{array}$$

Now we show that the σ defined in this way also satisfies the condition $\mathrm{Tr}(\sigma \sigma^{†})=1$. This is as follows.

$$\begin{array}{c} \mathrm{Tr}\left(\sigma \sigma^{†}\right)=\mathrm{Tr}\left(\frac{O-\langle O\rangle }{\Delta O}\rho^{\frac{1}{2}}{\left(\frac{O-\langle O\rangle }{\Delta O}\rho^{\frac{1}{2}}\right)}^{†}\right)\\ =\mathrm{Tr}(\left(\frac{O-\langle O\rangle }{\Delta O}\right)\rho^{\frac{1}{2}}\rho^{\frac{1}{2}}\left(\frac{O-\langle O\rangle }{\Delta O}\right))\\ =\mathrm{Tr}\left(\left(\frac{O-\langle O\rangle }{\Delta O}\right)\rho \left(\frac{O-\langle O\rangle }{\Delta O}\right)\right)=\mathrm{Tr}\left(\rho {\left(\frac{O-\langle O\rangle }{\Delta O}\right)}^{2}\right)=1 \end{array}$$

As a result, we have derived another set of operators σ that satisfies the required conditions essential for deriving the stronger quantum speed limit bound for mixed quantum states. Additionally, we see that since *O* can be any Hermitian operator, therefore we can have a large set of σ as stated above that satisfies our required criterion based on the different Hermitian operators that we can choose. Using this way of finding σ, the stronger quantum speed limit bound is simplified further, as follows. We start with the expression of R(t) which is as follows:

(38) $$\begin{array}{c} R\left(t\right)=\frac{1}{2}{\left|\mathrm{Tr}\left(\rho^{\frac{1}{2}}\left(\frac{A}{\Delta A}\pm i\frac{B}{\Delta B}\right)\sigma \right)\right|}^{2}. \end{array}$$

We put the expression of σ as described in this section and find the following expression for R(t):

(39) $$\begin{array}{c} R\left(t\right)=\frac{1}{2}{\left|\mathrm{Tr}\left(\rho^{\frac{1}{2}}\left(\frac{A}{\Delta A}\pm i\frac{B}{\Delta B}\right)\left(\frac{O-\langle O\rangle }{\Delta O}\rho^{\frac{1}{2}}\right)\right)\right|}^{2}. \end{array}$$

Using the cyclic property of the trace function, therefore, we arrive at the following simplified version of R(t):

(40) $$\begin{array}{c} R\left(t\right)=\frac{1}{2}{\left|\mathrm{Tr}\left(\rho \left(\frac{A}{\Delta A}\pm i\frac{B}{\Delta B}\right)\left(\frac{O-\langle O\rangle }{\Delta O}\right)\right)\right|}^{2}. \end{array}$$

□

The above expression is clearly computationally much more efficient and less time-consuming, where for the calculation of the stronger speed limit bound for mixed quantum states, one does not have to compute the square root of ρ, making the calculation of the bound more efficient, fast, and simple. We will apply this technique for the examples in the next section.

## 4. Examples

### 4.1. Random Hamiltonians

In this section, we calculate and compare the bound given by the tighter quantum speed limit bound with that of the MT-like bound of mixed-state generalization using random Hamiltonians from the Gaussian Unitary Ensemble, or GUE in short. Random Hamiltonians from GUE have found use in many different areas. However, our reason for choosing Hamiltonians randomly from GUE is that they give vaild Hamiltonians that are also diverse such that we can show the performance of our stronger quantum speed limit bound for mixed quantum states and unitary evolutions for diverse cases.

Mathematically, a random Hamiltonian is a D×D Hermitian operator *H* in D×D dimensional Hilbert space, drawn from a Gaussian unitary ensemble (GUE). The GUE is described by the following probability distribution function:

(41) $$\begin{array}{c} P\left(H\right)=Ce^{-\frac{D}{2}\mathrm{Tr}\left(H^{2}\right)} \end{array}$$

where *C* is the normalization constant and the elements of *H* are drawn from the Gaussian probability distribution. In this way, *H* is also Hermitian. A random Hamiltonian dynamic is a unitary time-evolution generated by a fixed time-independent GUE Hamiltonian.

**Figure 1:**
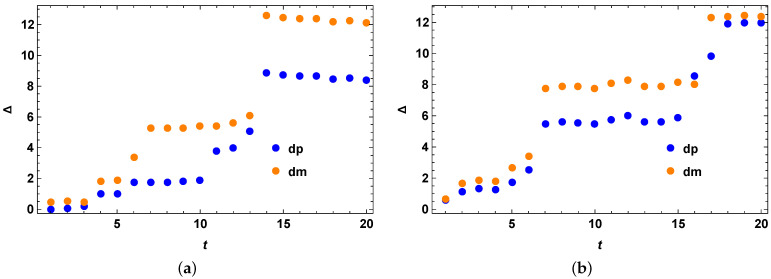
Random Hamiltonian examples. (**a**) Random Hamiltonian example 1 with a fixed initial mixed quantum state over full time range with no optimization over the Hermitian matrices *O*. The vertical axis represents $\Delta =\tau_{SQSL}-\tau_{MTL}$, for the case of both blue (when R(t) in Equation (15) has + sign inside) and orange (when R(t) in Equation (15) has − sign inside) data points. The horizontal axis *t* represents evolution time. (**b**) Random Hamiltonian example 2 with a fixed initial mixed quantum state over full time range with optimization over three Hermitian matrices *O*. The vertical axis represents $\Delta =\tau_{SQSL}-\tau_{MTL}$, for the case of both blue (when R(t) in Equation (15) has + sign inside) and orange (when R(t) in Equation (15) has − sign inside) data points. The horizontal axis *t* represents evolution time.

We take the Hilbert space of dimension 3 for our numerical example as shown in Figure 1. The initial state is taken as the following:

(42) $$\begin{array}{c} \rho_{0}=0.2|0\rangle \langle 0|+0.5|1\rangle \langle 1|+0.3|2\rangle \langle 2| \end{array}$$

Following the second method of generating appropriate σ using a set of Hermitian operators *O*, we obtain the quantum speed limit bound for the mixed quantum states. We compare the performance of our optimized bound with the previous bounds and non-optimized version of our bound as given in the figures. From both Figure 1a,b, we clearly see that our theory is correct and we have $\Delta =\tau_{SQSL}-\tau_{MT}$ as always positive, showing that the stronger quantum speed limit bound always outperforms the MT-like bound for mixed quantum states and unitary evolution. In Figure 1, at t=0, all the values of Δ are zero because all the random Hamiltonians start with being identity at t=0. All the Hamiltonians taken here are time-independent by construction. In Figure 1b, we perform an optimization over different sets of σ so as to get a better bound, whereas in Figure 1a, we still get good results even without any optimization. In the figures and everywhere in the later examples in the next sections, dp represents the difference of our bound with the MT-like bound, as in Equation (4) when one uses a + sign in front of R(t) and dm represents the difference of our bound with the MT-like bound, as in Equation (4) when one uses a − sign in front of R(t), unless stated otherwise. We also perform optimization of our bound over small sets of σ and note that our bound performs better with or without optimization in these cases, as exemplified by the figures. When we perform optimization, it is simple and easily completed within about a minute in most cases for such small sets of σ such as 5 or 10 number of σ as stated in the caption of the figures. This makes our method computationally practical and feasible. This simple optimization also gives noticeable improvement on the bounds as demonstrated by the figures, in this example as well as other examples in the following sections. However, since we cannot tell a priori which optimized version will give the best bound and in which region due to no closed form of the optimized version for arbitrary Hamiltonian, as a result we keep this as an open question for future investigation.

### 4.2. Anisotropic Multiqubit Heisenberg Spin Chain

A lot of attention has been devoted to the study of graph states, which act as an important and central resource in quantum error correction, quantum cryptography, and practical quantum metrology in the presence of noise. As a result, owing to its importance in quantum information processing tasks, we write here the entangling Hamiltonian of the graph state generation for the multiqubit case as follows.

(43) $$\begin{array}{c} H=\sum_{i=1}^{N}\lambda_{i}^{z}\sigma_{i}^{z}+\sum_{i=1}^{N}\lambda^{zz}\sigma_{i}^{z}\sigma_{i+1}^{z}-\\ \sum_{i=1}^{N}\lambda^{xx}\sigma_{i}^{x}\sigma_{i+1}^{x}-\sum_{i=1}^{N}\lambda^{yy}\sigma_{i}^{y}\sigma_{i+1}^{y} \end{array}$$

In terms of the experiments, the above Hamiltonian is used in the physical implementation of the optical lattice of ultracold bosonic atoms. This is also the anisotropic Heisenberg spin model in the optical lattice model which can be written down in the appropriate way using the creation and the annihilation operators. The Hamiltonian has the local terms as well as the interaction terms, and in general for *N* spins which can be mapped to *N* qubits. In general, the coefficients {λ} are time-dependent. However, for simplicity we take this to be time-independent in our case and calculate the quantum speed limit bound for evolution under this Hamiltonian for initially mixed quantum states.

**Figure 2:**
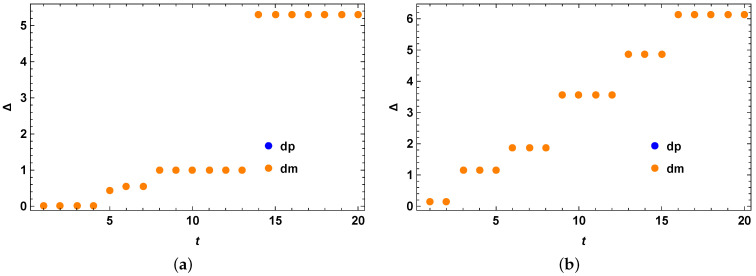
Anisotropic Heisenberg spin chain Example 1. (**a**) Anisotropic Heisenberg spin chain example 1 with a fixed initial mixed quantum state over full time range with no optimization over the Hermitian matrices *O*. The vertical axis represents $\Delta =\tau_{SQSL}-\tau_{MTL}$, for the case of both blue (when R(t) in Equation (15) has + sign inside) and orange (when R(t) in Equation (15) has − sign inside) data points. The horizontal axis *t* represents evolution time. Blue and orange dots coincide. (**b**) Anisotropic Heisenberg spin chain example 1 with a fixed initial mixed quantum state over full time range with optimization over three Hermitian matrices *O*. The vertical axis represents $\Delta =\tau_{SQSL}-\tau_{MTL}$, for the case of both blue (when R(t) in Equation (15) has + sign inside) and orange (when R(t) in Equation (15) has − sign inside) data points. The horizontal axis *t* represents evolution time. Blue and orange dots coincide.

We take the Hilbert space of dimension 4 for numerical example 1 as shown in Figure 2a,b, i.e., for the case of two qubits. The initial state is taken as the following:

(44) $$\begin{array}{c} \rho_{0}=0.7|0\rangle \langle 0|+0.1|1\rangle \langle 1|+0.1|2\rangle \langle 2|+0.1|3\rangle \langle 3| \end{array}$$

**Figure 3:**
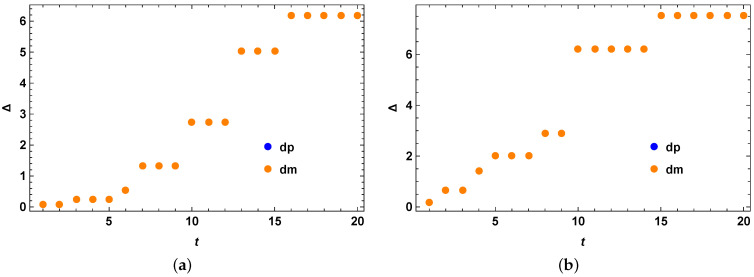
Anisotropic Heisenberg spin chain Example 2. (**a**) Anisotropic Heisenberg spin chain example 2 with a fixed initial mixed quantum state over full time range with no optimization over the Hermitian matrices *O*. The vertical axis represents $\Delta =\tau_{SQSL}-\tau_{MTL}$, for the case of both blue (when R(t) in Equation (15) has + sign inside) and orange (when R(t) in Equation (15) has − sign inside) data points. The horizontal axis *t* represents evolution time. Blue and orange dots coincide. (**b**) Anisotropic Heisenberg spin chain example 2 with a fixed initial mixed quantum state over full time range with optimization over three Hermitian matrices *O*. The vertical axis represents $\Delta =\tau_{SQSL}-\tau_{MTL}$, for the case of both blue (when R(t) in Equation (15) has + sign inside) and orange (when R(t) in Equation (15) has − sign inside) data points. The horizontal axis *t* represents evolution time. Blue and orange dots coincide.

Following the second method of generating appropriate σ, we obtain the quantum speed limit bound for the mixed quantum states. We check our bound for the initial mixed quantum state as above under the action of the anisotropic Heisenberg spin-chain Hamiltonian and compare the performance of our optimized bound with the previous bound. From Figure 2a,b, we clearly see that our theory is correct and we have $\Delta =\tau_{SQSL}-\tau_{MTL}$ as always positive, showing that the tighter quantum speed limit bound always outperforms the MT-like bound for mixed quantum states. The same holds for the example 2 as given in Figure 3a,b, where a different instance of the anisotropic Heisenberg spin has been considered with a different set of parameters but with the same underlying model as stated here. Since we cannot tell a priori which optimized version will give the best bound and in which region, as a result we keep this as an open question for future investigation.

### 4.3. Perfect State Transfer Hamiltonian

Here, we take the example of a Hamiltonian which is useful for the case of perfect quantum state transfer, as quantum state transfer is one of the important quantum information processing tasks. The Hamiltonian describing the case of perfect state transfer is given by the following:

(45) $$\begin{array}{c} H=\sum_{n=1}^{N-1}J_{n}\sigma_{n}^{z}\sigma_{n+1}^{z}+\sum_{n=1}^{N}B_{n}\sigma_{n}^{x}, \end{array}$$

where *N* is the number of qubits. As specific numerical examples, we take the Hilbert space of dimension 4, i.e., for the case of two qubits. In this case, we take $J_{k}=\frac{1}{2},B_{k}=\frac{1}{2}$ and then the Hamiltonian reads as the following for the case of two qubits as:

(46) $$\begin{array}{c} H=J_{1}\left(\sigma^{z}⊗\sigma^{z}\right)+B_{1}\left(\sigma^{x}⊗I\right)+B_{2}\left(I⊗\sigma^{x}\right). \end{array}$$

The initial state is taken as the following:

(47) $$\begin{array}{c} \rho_{0}=0.7|0\rangle \langle 0|+0.1|1\rangle \langle 1|+0.1|2\rangle \langle 2|+0.1|3\rangle \langle 3|. \end{array}$$

**Figure 4:**
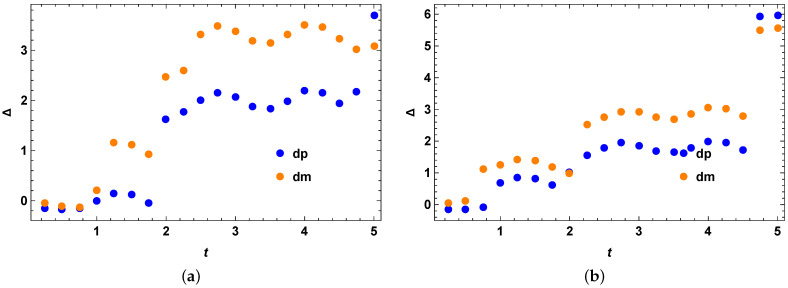
Perfect state transfer Hamiltonian example. (**a**) Perfect state transfer Hamiltonian evolution of an initial mixed quantum states in two qubit Hilbert space with no optimization over random Hermitian operators *O*. The vertical axis represents $\Delta =\tau_{SQSL}-\tau_{MTL}$, for the case of both blue (when R(t) in Equation (15) has + sign inside) and orange (when R(t) in Equation (15) has − sign inside) data points. The horizontal axis *t* represents evolution time. (**b**) Perfect state transfer Hamiltonian evolution of an initial mixed quantum states in two qubit Hilbert space with optimization over 3 random Hermitian operators *O*. The vertical axis represents $\Delta =\tau_{SQSL}-\tau_{MTL}$, for the case of both blue (when R(t) in Equation (15) has + sign inside) and orange (when R(t) in Equation (15) has − sign inside) data points. The horizontal axis *t* represents evolution time.

We obtain the quantum speed limit bound for the mixed quantum states in a similar procedure as the other examples stated before. We check our bound for the initial mixed quantum state as stated above under the action of the quantum walker Hamiltonian as stated before and compare the performance of our optimized bound with the previous MT-like bound for mixed quantum states. From Figure 4a,b, we clearly see that our theory is correct and we have $\Delta =\tau_{SQSL}-\tau_{MTL}$ as always positive, showing that the tighter quantum speed limit bound always outperforms the MT-like (MTL) bound for mixed quantum states.

### 4.4. Hamiltonian Evolution of a Separable State

**Figure 5:**
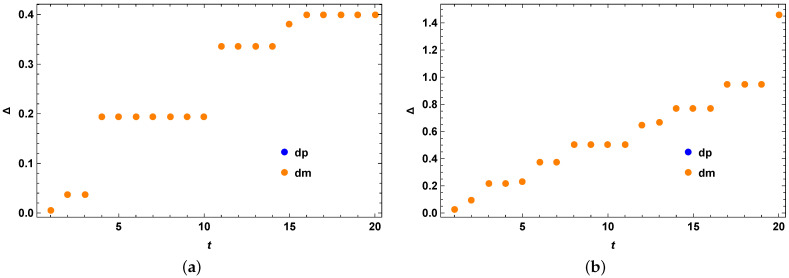
Hamiltonian evolution of initial separable mixed quantum state according to Hamiltonian in Equation (48). (**a**) Hamiltonian evolution of an initial separable mixed quantum states in two qutrit Hilbert space, according to Hamiltonian in Equation (48), without any optimization over random Hermitian operators *O*. The vertical axis represents $\Delta =\tau_{SQSL}-\tau_{MTL}$, for the case of both blue (when R(t) in Equation (15) has + sign inside) and orange (when R(t) in Equation (15) has − sign inside) data points. The horizontal axis *t* represents evolution time. The blue and the orange dots coincide here. (**b**) Perfect state transfer Hamiltonian evolution of an initial mixed quantum states in two qubit Hilbert space, according to Hamiltonian in Equation (48), with optimization over 5 random Hermitian operators *O*. The vertical axis represents $\Delta =\tau_{SQSL}-\tau_{MTL}$, for the case of both blue (when R(t) in Equation (15) has + sign inside) and orange (when R(t) in Equation (15) has − sign inside) data points. The horizontal axis *t* represents evolution time. The blue and the orange dots coincide here.

Here, we take the example of another type of Hamiltonian which drives the evolution of an initially mixed quantum state which we take to be a separable quantum state. The Hamiltonian describing this case is given by the following:

(48) $$\begin{array}{c} H=\sum_{i=1}^{M}H_{i}\;\;\;,\;\;\;H_{i}=\omega \hbar \sum_{n=0}^{N-1}n|n\rangle \langle n| \end{array}$$

where *M* is the number of qubits and *N* is the dimension of each subsystem. As we mentioned, we take the initial state as a separable mixed state. This choice bears no particular importance. For our case of numerical example, we take the case of a quantum system of two qutrits. Even for this case of two qutrits, the derivation of the stronger quantum speed limit for mixed states is done within a fraction of a minute, even for an optimization over a set of 5 number of σ operators. This implies that the derivation of the quantum speed limit for mixed quantum states can be done for a wide variety of quantum systems of different dimensions, in this case the dimension being 9. We demonstrate here a particular example by taking the following initial quantum state:

(49) $$\begin{array}{c} \rho_{0}=a|0\rangle \langle 0|+b|1\rangle \langle 1|+c|2\rangle \langle 2|+\left(1-a-b-c-d-e\right)|3\rangle \langle 3|+d|7\rangle \langle 7|+e|8\rangle \langle 8|, \end{array}$$

where we have the following parameters, a=0.175,b=0.25,c=0.15,d=0.105, and e=0.255. We have also set ωℏ=1 without any loss of generality. The choice of these parameters is arbitrary. A different choice of these parameters does not bear any effect on the computational complexity of the stronger quantum speed limit bound for mixed quantum states. Next, we obtain the quantum speed limit bound for the mixed quantum states in the same procedure as the other examples mentioned before. We plot our results in Figure 5 From this figure, we again see that our theory give good improvement over the previous MTL quantum speed limit bound and we have $\Delta =\tau_{SQSL}-\tau_{MTL}$ as always positive. The apparent difference in various points can be attributed to the fact that we always choose a random eigenbasis for the calculation of our bound.

### 4.5. Two Qubit CNOT Hamiltonian

The two qubit CNOT gate is an important case of a Hamiltonian as this is a part of the universal gates that can be used for performing all sorts of quantum computation. Therefore, we choose a Hamiltonian that will represent a two qubit CNOT gate. The form of one such Hamiltonian, also called the principal Hamiltonian, is given by $H=\pi \sigma_{z}^{-}⊗\sigma_{x}^{-}$ where we have used the following notation:

(50) $$\begin{array}{c} \sigma_{z}^{\pm }=\frac{I\pm \sigma_{z}}{2},\;\;\sigma_{x}^{\pm }=\frac{I\pm \sigma_{x}}{2}. \end{array}$$

**Figure 6:**
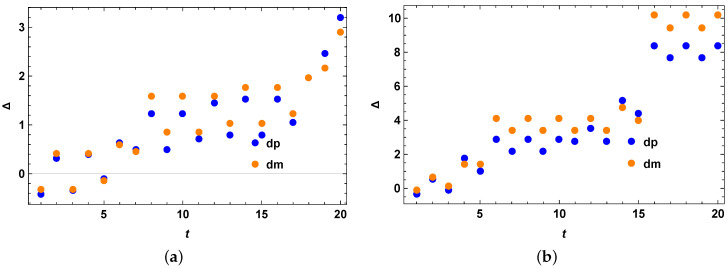
Two qubit CNOT gate Hamiltonian example. (**a**) Two qubit CNOT gate Hamiltonian evolution of an initial mixed quantum states in two qubit Hilbert space with no optimization over random Hermitian operators *O*. The vertical axis represents $\Delta =\tau_{SQSL}-\tau_{MTL}$, for the case of both blue (when R(t) in Equation (15) has + sign inside) and orange (when R(t) in Equation (15) has − sign inside) data points. The horizontal axis *t* represents evolution time. (**b**) Two qubit CNOT gate Hamiltonian evolution of an initial mixed quantum states in two qubit Hilbert space with optimization over 5 random Hermitian operators *O*. The vertical axis represents $\Delta =\tau_{SQSL}-\tau_{MTL}$, for the case of both blue (when R(t) in Equation (15) has + sign inside) and orange (when R(t) in Equation (15) has − sign inside) data points. The horizontal axis *t* represents evolution time.

We calculate the quantum speed limit bound for evolution under this Hamiltonian for initially mixed quantum states. We take the Hilbert space of dimension 4 for our numerical example as represented in Figure 6a,b, i.e., for the case of two qubits. The initial state is taken as the following:

(51) $$\begin{array}{c} \rho_{0}=0.7|0\rangle \langle 0|+0.1|1\rangle \langle 1|+0.1|2\rangle \langle 2|+0.1|3\rangle \langle 3| \end{array}$$

As with all the examples before, we calculate the stronger quantum speed limit bound using the same methods. We check our bound for the above choices of initial mixed quantum state and the Hamiltonian and compare the performance of our optimized bound with the previous bound. The optimization is over 10 such operators σ as in all the above cases. From the figure, we clearly see that we always have $\Delta =\tau_{SQSL}-\tau_{MTL}$ as positive, showing that the stronger quantum speed limit bound derived in this article outperforms the MT-like (MTL) bound for mixed quantum states. Additionally, it is natural to expect that our stronger speed limit bound will outperform the MT-like bound for mixed quantum states even better when the optimization is performed over a larger set of σ.

### 4.6. Comparison with Other Bounds: Perfect State Transfer Hamiltonian

**Figure 7:**
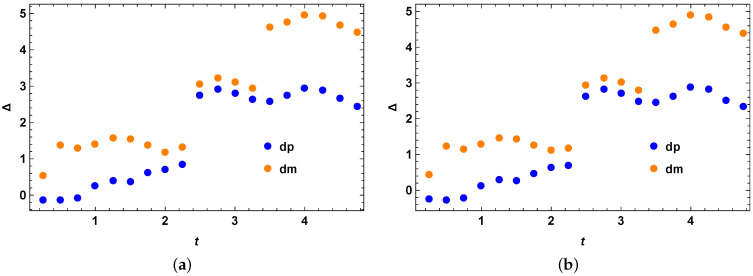
Difference with the third existing quantum speed limit bound with our stronger quantum speed limit bound for the perfect state transfer Hamiltonian. (**a**) Difference with the second existing quantum speed limit bound with our stronger quantum speed limit bound for the perfect state transfer Hamiltonian with optimization over 5 random Hermitian operators *O*. The vertical axis represents $\Delta =\tau_{SQSL}-\tau_{PRE1}$, where $\tau_{PRE1}$ is given by Equation (5), for the case of both blue (when R(t) in Equation (15) has + sign inside) and orange (when R(t) in Equation (15) has − sign inside) data points. The horizontal axis *t* represents evolution time. It looks very similar to the next one, but there is a small difference which is shown in the next plot. (**b**) Difference with the third existing quantum speed limit bound with our stronger quantum speed limit bound for the perfect state transfer Hamiltonian with optimization over 5 random Hermitian operators *O*. The vertical axis represents $\Delta =\tau_{SQSL}-\tau_{PRE2}$, where $\tau_{PRE2}$ is given by Equation (8), for the case of both blue (when R(t) in Equation (15) has + sign inside) and orange (when R(t) in Equation (15) has − sign inside) data points. The horizontal axis *t* represents evolution time. It looks very similar to the previous one, but there is a small difference which is shown in the next plot.

**Figure 8:**
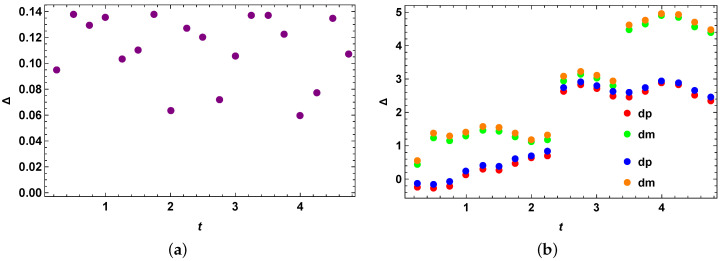
Difference with the stronger speed limit bound for plus sign in R(t) with the stronger speed limit bound for minus sign in R(t) for mixed quantum states for the perfect state transfer Hamiltonian from the second and the third previous quantum speed limit bounds. (**a**) Difference between the ‘difference between second existing quantum speed limit bound with our stronger quantum speed limit bound and the difference between third existing quantum speed limit bound with our stronger quantum speed limit bound’ for the perfect state transfer Hamiltonian for minus sign in R(t). The vertical axis represents $\Delta =\left(\Delta_{SQSL}-\Delta_{PRE1}\right)-\left(\Delta_{SQSL}-\Delta_{PRE2}\right)=\left(\Delta_{PRE2}-\Delta_{PRE1}\right)$. The horizontal axis represents the evolution time. (**b**) Difference between the stronger speed limit bound using plus sign in R(t) and the stronger speed limit bound using minus sign in R(t) for mixed quantum states for the perfect state transfer Hamiltonian from the second and the third previous quantum speed limit bounds. The vertical axis represents $\Delta =(\Delta_{SQSL}-\Delta_{PRE1})$ or $\left(\Delta_{SQSL}-\Delta_{PRE2}\right)$ as represented by different colours explained in Section 4.6. The horizontal axis represents the evolution time.

Here, we take the example of perfect quantum state transfer for comparing our stronger quantum speed limit bound for mixed quantum states with two other existing important bounds for the quantum speed limit for mixed quantum states. The Hamiltonian describing this case is given by Equations (45) and (46), and the initial quantum state as given by Equation (47). We obtain the quantum speed limit bound for the mixed quantum states in a similar way as before and compare the performance of our optimized bound with the previous two quantum speed limit bound for mixed quantum states as given in [41]. Note that the quantum speed limit bounds given in [41] are better than the MT-like bounds for most qubit states. We check from Figure 7a,b that our bound is better than the second and the third existing quantum speed limit bounds as given in [41], in these cases with the minimum number of optimizations as stated in their respective figures. The optimization is simple and minimal and is completed within about a minute for five optimizations. As a result, this optimization is highly practical and feasible. We notice that Figure 7a,b look almost identical. As a result, we check whether they are actually numerically identical or there is a difference between them. We plot the difference between the second and third quantum speed limit bounds as given in the paper [41] and plot it in Figure 8a, which shows that they are actually different by a small margin. Next, we check whether the + and − signs in front of R(t) in our stronger quantum speed limit bounds make a difference in our stronger quantum speed limit bounds. We again choose the perfect state transfer Hamiltonian as before and plot these bounds as represented in Figure 8b. As explained in the Figure 8b, we see that there are differences with the stronger speed limit bound for the plus sign in R(t) with the stronger speed limit bound for the minus sign in R(t) for mixed quantum states for the perfect state transfer Hamiltonian from the second and the third previous quantum speed limit bounds as given in the paper [41]. dp represents the difference of our bound with the second (blue) and the third (red) when one uses a + sign in front of R(t) in Equation (15) and dm represents the difference of our bound with the second (orange) and the third (green) when one uses a − sign in front of R(t) in Equation (15), which highlights all the essential differences between these bounds. This plot also demonstrates that our bound, represented by Equation (15), performs better than the previous bounds for both the cases of + and − signs in front of R(t).

## 5. Discussion

In this work, we have derived a stronger quantum speed limit for mixed quantum states using the mixed state generalization of stronger preparation uncertainty relations. We have shown that this bound reduces to that of the pure states under appropriate conditions. Thereafter, we have discussed methods to derive the suitable operators that allows us to calculate our bound. Hereafter, we have shown numerically using random Hamiltonians obtained from a Gaussian Unitary ensemble, that our bound performs better than the mixed state version of the MT bound. The reason for taking random Hamiltonians is nothing but that the technique provides valid Hamiltonians that are unlike each other. Additionally, we have then shown, using many suitable analytical examples of Hamiltonians useful in quantum information and computation tasks, that the stronger quantum speed limit bound derived here for mixed quantum states also performs better than the MT-like bound and also two more existing quantum speed limit bounds for mixed quantum states existing in the current literature. Future directions remain open for comparing our bound to those of other bounds in the literature for mixed quantum states.

## Data Availability

The data that support the findings of this study are available from the corresponding author upon reasonable request.
